# Utility of urine culture in men with uncomplicated cystitis in ambulatory settings

**DOI:** 10.1017/ash.2025.42

**Published:** 2025-02-27

**Authors:** Mackenzie R. Keintz, Bryan T. Alexander, Katherine Fagan, Trevor C. Van Schooneveld

**Affiliations:** 1 Division of Infectious Diseases, University of Nebraska Medical Center, Omaha, NE, USA; 2 Department of Pharmaceutical & Nutrition Care, Nebraska Medicine, Omaha, NE, USA; 3 Analytics Team, Nebraska Medicine, Omaha, NE, USA

## Abstract

Urinary tract infections (UTI) are common and women with typical symptoms may be treated empirically without culture. We evaluated the utility of urine culture in men presenting with typical symptoms of uncomplicated UTI. Most pathogens isolated retained susceptibility to first-line antimicrobials suggesting urine cultures may be unnecessary in this population.

## Introduction

Urinary tract infections (UTIs) are common, accounting for about 10.2 million ambulatory care visits annually in the United States.^1,2^ Although less common, about 20% of UTIs occur in men, with risk increasing with age and comorbidity.^3^ In healthy premenopausal women presenting with typical UTI symptoms, current guidelines do not recommend urine cultures prior to initiating first-line empiric antibiotics. Historically, men have been excluded from a diagnosis of uncomplicated UTI; however, more recent data suggests that male patients can be treated with shorter courses of antibiotics similarly to female patients in the absence of signs or symptoms of prostatitis.^4–6^ In this population, urine cultures are typically used to guide therapy in complicated UTIs, although little literature exists on this topic. Guidelines currently do not provide any specific recommendation for male patients to receive a urine culture in the presence of classic uncomplicated urinary symptoms.

First-line recommended agents for uncomplicated UTI are nitrofurantoin or trimethoprim/sulfamethoxazole, regardless of gender. Oral beta-lactams are considered an alternative therapy, and broad-spectrum antimicrobials including fluoroquinolones are no longer routinely recommended due to their side effect profile and risk of inducing antimicrobial resistance.^7^

We created an internal quality metric to measure appropriate prescribing for uncomplicated UTIs using coding data, which evaluates the use of first- or second-line UTI therapy.^8^ This metric includes men, which have traditionally been considered complicated UTI, and for which urine culture is therefore often assumed to be necessary for directing therapy. Thus, we sought to evaluate the activity of first- and second-line antimicrobial agents in men with uncomplicated UTIs to determine the utility of urine culture in this population and the validity of our UTI metric.

## Methods

We included male patients diagnosed as having uncomplicated UTI between January 1, 2021, and June 30, 2024, with a positive urine culture in a tertiary healthcare system-affiliated primary or immediate care clinic. We utilized ICD-10 codes to identify clinic visits secondary to UTI including N30.00, N30.01, N30.80, N30.81, N30.90, N30.91, N34.1, N34.2, N34.3, N39.0, and N39.9. A chart review was performed to include only patients presenting with typical symptoms of cystitis documented in the visit including dysuria, frequency, and urgency. Patients were excluded if they had symptoms of complicated UTI including fever, costovertebral angle tenderness, signs or symptoms of prostatitis, indwelling Foley or suprapubic catheter, or recurrent UTI (defined as >2 UTI in the past 6 months OR >3 UTIs in the past 12 months) based on chart review. Additionally, patients were excluded if they had ICD-10 codes for renal transplant, urinary catheter, neutropenia, or additional ICD-10 codes for non-urinary infections that would require an antibiotic. Urine cultures were considered positive if they identified at least one organism with >100,000 colony-forming units. Contaminated cultures were excluded and defined as identifying >3 organisms present. We evaluated organisms from positive urine cultures for susceptibility to nitrofurantoin, trimethoprim/sulfamethoxazole, or cefazolin as a surrogate for oral beta-lactams. Fosfomycin susceptibility was not evaluated as it is not routinely performed at our institution. First-line antibiotic for UTI was defined as the use of nitrofurantoin or trimethoprim/sulfamethoxazole, and second-line antibiotic use included oral beta-lactams, based on current Infectious Diseases Society of America guidelines for acute uncomplicated UTI. All microorganisms were included in the analysis regardless of inherent resistance to first-line or second-line antibiotics.

## Results

Ninety-seven episodes of uncomplicated cystitis with typical symptoms were identified in male outpatients during the study period. The median age of included patients was 64 (IQR 54.5, 75.5). More patients were seen in immediate care clinics than in primary care clinics (58% vs 42%). The most common organism detected was *Escherichia coli (54%)*, with a further 25% comprised of other Enterobacterales (Table 1).

**Table 1.** Patient and urinary pathogen characteristics and susceptibilities

Characteristic	Isolates (N=97)
Age, median (IQR)	64 (54.5, 75.5)
Clinic setting, percent (n)	
Primary care	42% (41/97)
Immediate care	58% (56/97)
Bacteria, percent (n)	
*Escherichia coli*	54% (52/97)
*Staphylococcus* spp.	9% (9/97)
*Enterococcus faecalis*	7% (7/97)
*Klebsiella pneumoniae*	6% (6/97)
*Proteus mirabilis*	6% (6/97)
*Enterobacter cloacae*	4% (4/97)
*Klebsiella oxytoca*	4% (4/97)
*Citrobacter koseri*	2% (2/97)
*Klebsiella variicola*	2% (2/97)
*Morganella morganii*	2% (2/97)
*Klebsiella aerogenes*	1% (1/97)
*Pseudomonas aeruginosa*	1% (1/97)
*Streptococcus agalactiae*	1% (1/97)
Outcomes, percent (n)	
Susceptible to at least one first-line agent	99% (96/97)
Susceptible to both first-line agents	71% (69/97)
Susceptible to nitrofurantoin	84% (81/97)
Susceptible to TMP/SMX	87% (84/97)
Susceptible to cefazolin	73% (71/97)

Only one isolated organism was identified as resistant to both first-line antibiotics, a *Pseudomonas aeruginosa* isolate. Seventy-one percent of isolated organisms were susceptible to both first-line antibiotics, with high rates of susceptibility to both nitrofurantoin (84%) and trimethoprim/sulfamethoxazole (87%). Beta-lactams also retained a relatively high rate of activity with 73% of organisms susceptible (Table 1).

## Discussion

Our study revealed the majority of urinary pathogens isolated from men presenting with uncomplicated UTI exhibiting typical symptoms retained susceptibility to first-line antimicrobials. These findings have 2 implications. First, they suggest that it may be unnecessary to perform routine urine cultures in this patient population, similarly to their premenopausal female counterparts. They also validate our assessment of appropriate therapy to include only these first-line agents and suggest our hypothesis that the appropriate therapy choice identical between men and women is valid. Additionally, this finding highlights that guideline recommendations for first-line empiric antibiotic choices remain optimal choices for both men and women presenting with uncomplicated cystitis.

Our microbiologic findings were generally congruent with existing studies of male UTIs. *E. coli* is the predominant pathogen in women making up >90% of pathogens, but men show a more varied pathogen make-up.^9^ Pathogen detection varies by both study methodology and patient presentation. A large study in Germany of all positive male outpatient urine cultures documented *E. coli* in only 42%, while *Enterococcus faecalis* was found in 16% and *P. aeruginosa* in 5.5%.^10^ Similarly, a randomized trial of treatment of afebrile UTI in men found *E. coli* in 42% with *Klebsiella* species in 16% and *E. faecalis* in 9%.^4^ Contrasting this, *E. coli* and to a lesser extent *Klebsiella* species make up >85% of isolates in febrile UTI. The differences in these studies likely relate to varying definitions of UTI, with the likely or stated inclusion of asymptomatic bacteriuria, and recurrent or complicated UTI in the large population-based studies.

Our patient population had a lower rate of infection caused by *E. coli* than has been established in previous studies of women.^9^ Despite this difference, the first-line antimicrobials recommended by current guidelines remained highly clinically active and remain an acceptable standard for appropriateness in our stewardship metric. Current guidelines suggest that trimethoprim/sulfamethoxazole should not be used as an empiric treatment for acute uncomplicated UTI if resistance rates are >20% in female patients. This standard may be applied to the male population and may limit the generalizability of our findings.

One limitation was the single-center nature of our study, although it included a system with >250,000 primary care visits per year. We also noted relatively low antimicrobial resistance rates for first-line agents, which may not be true nationwide. Our study also included only men in which urine cultures were obtained, which will miss patients who were treated empirically and perhaps not fully represent the pathogen mix of the full male uncomplicated UTI population. These findings only apply to the population they were studied in and should not be applied to more complex infectious such as pyelonephritis, patients with stones, or those with indwelling urinary catheters.

In conclusion, our findings support the practice of not obtaining urine cultures for men with uncomplicated UTIs who respond well to first-line antimicrobials. Nevertheless, the applicability of this approach should be considered with caution in regions with higher rates of antimicrobial resistance or an alternative pathogen mix, necessitating local surveillance data to guide clinical decision-making.
